# Thiol- and Biotin-Labeled Probes for Oligonucleotide Quartz Crystal Microbalance Biosensors of Microalga *Alexandrium Minutum*

**DOI:** 10.3390/bios2030245

**Published:** 2012-07-03

**Authors:** Mathieu Lazerges, Hubert Perrot, Niriniony Rabehagasoa, Chantal Compère

**Affiliations:** 1 Centre National de la Recherche Scientifique (CNRS), Unité Propre de Recherche (UPR) 15, Laboratoire Interfaces et Systèmes Electrochimiques, 4 place Jussieu, Paris 75252, France; E-Mails: hubert.perrot@upmc.fr (H.P.); niriniony.rabehagasoa@ird.fr (N.R.); 2 Université Pierre et Marie Curie, LISE, 4 place Jussieu, Paris 75252, France; 3 Institut Français de Recherche pour l’Exploitation de la Mer (IFREMER), Centre de Brest Service Interfaces et Capteurs, Z.I. pointe du diable, Plouzané 29280, France; E-Mail: chantal.compere@ifremer.fr

**Keywords:** *Alexandrium Minutum*, avidin, biosensor, biotin, DNA, quartz crystal miocrobalance, self-assembled monolayer, thiol

## Abstract

Two quartz crystal microbalance oligonucleotide biosensors of a toxic microalga gene sequence (*Alexandrium Minutum*) have been designed. Grafting on a gold surface of 20-base thiol- or biotin-labeled probe, and selective hybridization with the complementary 20-base target, have been monitored *in situ* with a 27 MHz quartz crystal microbalance under controlled hydrodynamic conditions. The frequency of the set up is stable to within a few hertz, corresponding to the nanogram scale, for three hour experiments. DNA recognition by the two biosensors is efficient and selective. Hybridization kinetic curves indicate that the biosensor designed with the thiol-labeled probe is more sensitive, and that the biosensor designed with the biotin-labeled probe has a shorter time response and a higher hybridization efficiency.

## 1. Introduction

Quartz crystal microbalance (QCM), usually based on a thickness shear mode resonator [1], is useful to design sensitive and selective *in situ* time resolved DNA-biosensors [2,3,4]. QCM DNA-biosensors are used in various fields of human health: genetic diagnosis [5,6,7], detection of genetically modified organisms [8], bacteria detection [9] and toxicology [10]. A QCM DNA-biosensor for a specific gene sequence of a harmful microalga (*Alexandrium minutum*) [11,12,13] which produces paralytic shellfish poisoning (PSP) [14] is designed herein. Harmful algal blooms of this toxic algae are responsible for marine production (*i.e.*, shellfish farming) health damage [15,16,17]. Biosensor design is interesting in this case as current analytical methods used for coastal monitoring of toxic algae are time consuming and require animal experiments and taxonomists. An easy and direct method to design a DNA-biosensor consists of chemical adsorption of a thiol or disulfide labeled DNA probe onto a gold covered quartz surface [5,8,18]. Amine-labeled DNA probes can be grafted via EDC/NHS activation onto a gold surface covered with a mercaptopropanoic acid SAM [18]. Another grafting pathway is the complex formation of a biotin-labeled DNA probe with an avidin self-assembled monolayer deposited on a gold covered quartz surface [18]. These biosensor schemes are based on a self-assembled monolayer on a gold substrate, which is very stable to chemical oxidation and permits the coupling of acoustic detection with electrochemical measurements [19,20,21]. Comparative hybridization behavior of two QCM DNA-biosensors, based on both thiol- and biotin-labeled probes on a gold surface, are presented in this work. The 20-base DNA target of the biosensor is a partial sequence of the gene encoding for the large ribosomal RNA sub-unit of the *Alexandrium minutum*.

## 2. Experimental Section

### 2.1. Chemical and Biochemical Reagents

H_2_SO_4_ (95%), H_2_O_2_ (30%), MgCl_2_, NaOH, HCl, NaCl, 1 M hydroxyethylpiperazine-ethanesulfonic acid (HEPES), white egg avidin, dithiopropionic acid, 1 M tris-buffered saline (TRIS), sodium dodecyl sulfate (SDS), sodium citrate, casein, sarkosyl, ethylcarbodiimide (EDC) and N-hydroxysuccinimide (NHS) were purchased from Sigma Aldrich (biochemical grade). The blocking reagent for hybridization on nylon membranes was supplied by Roche. Water used was deionized and double distilled. DNA strands were obtained from Eurogentec [22] (Table 1).

**Table 1:** DNA probes (P), complementary targets (T) and random strand (R) structures.

Name	Sequence
P1	5’ AGCAC TGATG TGTAA GGGCT 3’
P2	thiol C6 5’ AGCAC TGATG TGTAA GGGCT 3’
P3	biotin C6 5’ AGCAC TGATG TGTAA GGGCT 3’
T1	3’ TCGTG ACTAC ACATT CCCGA 5’ phosphatase
T2	3’ TCGTG ACTAC ACATT CCCGA 5’
R	3’ CCTTG GTCTG TGTTT CAAGA 5’

Purity of DNA was checked by chromatography and MALDI-TOF analysis. DNA was quantified by UV optical density measurements.

### 2.2. Buffer and Solutions

The buffer for hybridization on nylon membranes used was 0.6 M NaCl, 60 mM sodium citrate, 1% blocking reagent, 0.1% sarkosyl, 0.02% SDS, pH 7.0. Post-hybridization saline-sodium citrate (SSC) washing solutions were prepared from 3 M NaCl, 300 mM sodium citrate, pH 7.2 and 10% SDS (20× SSC). Tris buffer for the color test used was 0.1 M Tris, 0.5 mM MgCl_2_, pH 9.5. The solution for chemical adsorption on gold covered quartz surface of dithioproionic acid and thiol-labeled probe was NaCl 0.5 M solution. The biosensor hybridization solution was 0.05 M HEPES buffer, 0.5 M NaCl, pH 7.2 [5] and biosensor dehybridization solution was 0.5 M NaOH, 3 M NaCl.

### 2.3. Hybridization Protocol on Nylon Membrane

50 µL of 100 µM solution in sterile distilled water of *Alexandrium Minutum* complementary probes P1 (non-labeled), P2 (thiol-labeled) and P3 (biotin-labeled), and random probe (R), were spotted on a positively charged nylon membrane [23] using a Schleicher&Schuell filtration manifold. The membrane was let to dry and cross-linked by UV exposition for 3 min. Prior to hybridization, 2 µL of 50 µM complementary target were labeled with deoxyuridine triphosphate (dUTP) according to the digoxigenin (DIG) oligonucleotide tailing kit, and diluted with 10 mL hybridization buffer, to give a 10 nM final concentration. The hybridization steps were 30 min at 37 °C for prehybridization, 2 h at 37 °C for hybridization and followed by four stringency washing steps: 2 times over 5 min at room temperature with 2× SSC, 0.1% SDS and 2 times over 15 min at 37 °C with 0.1× SSC, 0.1% SDS. DIG-labeled hybrids were detected with a 150 µU/mL anti-DIG-alkaline phosphatase conjugate, together with the substrates nitro blue tetrazolium (NBT) and bromo-chloro-indolyl phosphate (BCIP), which give a light-blue precipitate. Just prior to use, 1 mL of 30 mg/mL NBT and 1 mL of 15 mg/mL BCIP were mixed in 100 mL of TRIS buffer. The nylon membrane was immersed in the color development solution. BCIP and NBT are two colorless substrates, which form a redox system. BCIP is oxidized by alkaline phosphatase to indigo by release of a phosphate group. NBT is reduced to diformazan. The reaction products formed a water insoluble dark blue precipitate on the membrane.

### 2.4. Quartz Crystal and Probes Grafting

AT-cut planar quartz crystals 14 mm diameter with a 9 MHz nominal resonance frequency (Matel Fordhal France) were used. Two identical gold electrodes, 200 nm thick and 5 mm in diameter, were deposited by evaporation techniques on both crystals sides, with a 25 nm chromium underlayer. The gold side used in the experiments was cleaned with a 10 µL drop of 50% H_2_SO_4_, 50% H_2_O_2_ for 30 min and rinsed with deionized double distilled water. Quartz for thiol-labeled probe grafting was used directly. Quartz used to graft the biotin-labeled probe was first covered with an avidin monolayer. A dithioproionic acid self-assembled monolayer was first deposited on the gold surface by circulation over one hour with a 1 mM dithiopropionic acid aqueous solution. The dithiopropionic carbonyl group was then activated by formation of an ester by circulation over 30 min. with a 1/1 100 mg/mL EDC ethanolic solution/100 mg/mL NHS aqueous solution. Avidin was grafted with a peptide bond onto the activated carbonyl groups in a third and last step by circulation over 1 h with a 0.1 mg/mL avidin HEPES solution (pH 7.2).

### 2.5. Microbalance Apparatus

The quartz crystals were connected with a silver conducting paste, through wires, to a BNC adaptator. A lab-made oscillator was designed to drive the crystal at 27 MHz, which corresponds to the third overtone of a 9 MHz quartz resonator. To improve the stability, all the electronic oscillator components were temperature-controlled by a Watlow heater current monitor with stability better than 0.1 °C. A lab-made cell was developed: the crystal was mounted between two O-ring seals inserted in a plexiglass cell. One face of the quartz was in contact with the solutions. The cell volume was 50 µL. The apparatus included a P1 micropump (Pharmacia) to assure a 50 µL/min constant flow of the solutions. The experiments were performed at 23 ± 1 °C, room temperature. The experimental QCM setup, constituted of the 27 MHz QCM and a frequency counter PM 6685, was computer controlled with a home-made C language software.

## 3. Results and Discussion

### 3.1. Hybridization on Nylon Membranes

**Figure 1:**
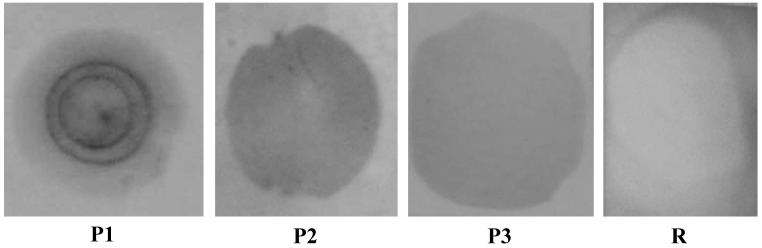
Hybridization colorimetric test on nylon membranes of *Alexandrium Minutum* complementary probes P1 (non labeled), P2 (thiol-labeled), P3 (biotin-labeled) and non complementary DNA R (random) with T1 complementary target (phosphatase-labeled).

Hybridization experiments are first performed on nylon membranes in order to check hybridization of thiol- and biotin-labeled probes. These tests are performed with random probe (R), and *Alexandrium Minutum* probes P1, P2 (thiol-labeled) and P3 (biotin-labeled). Hybridization nylon membrane colorimetric tests with phosphatase-labeled complementary target (T1) are presented in Figure 1.

The colorimetric test is positive for *Alexandrium Minutum* probes P1 (non labeled), P2 (thiol-labeled) and P3 (biotin-labeled) and negative for non complementary DNA R (random). These results indicate successful hybridization of thiol- and biotin-labeled probes.

### 3.2. Biosensor Designed with Thiol-Labeled Probe

**Figure 2:**
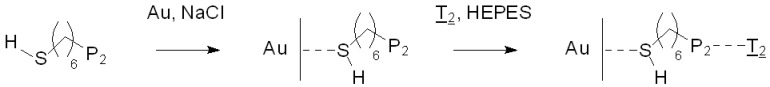
Thiol-labeled probe (P2) grafting and complementary target (T2) hybridization.

Formation of a self-assembled monolayer of thiol-labeled probe (P2) and hybridization recognition of the complementary target (T2) were performed on the gold covered face of a quartz resonator (Figure 2).

**Figure 3:**
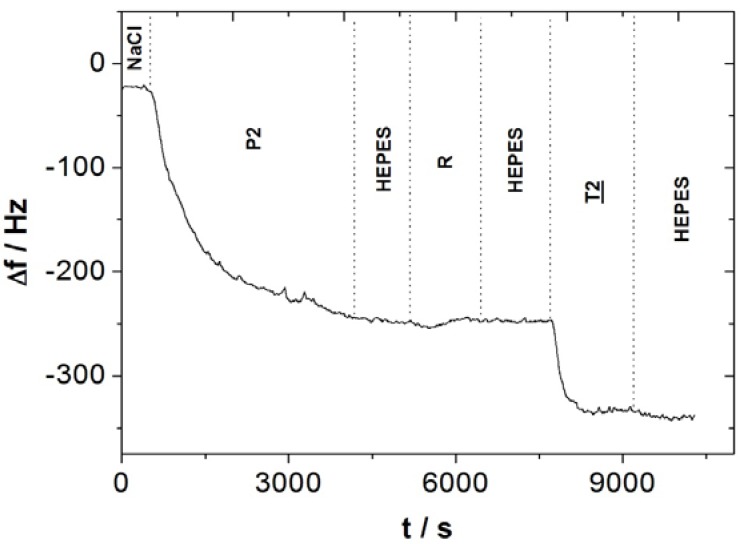
Microbalance frequency kinetics curves of probe (P2) grafting and selective complementary target (T2) recognition.

Frequency was monitored during the grafting and hybridization steps (Figure 3).

The first step, probe grafting, was performed by flowing of a 20 µg/mL probe (P2) NaCl solution. The frequency change measured during probe grafting was −225 Hz. The probe P2 surface density Γ_P1_ (probe/cm^2^) is:

Γ_P2_ = |Δf|∙s∙N_A_/S∙M_P2_       (1)

Γ_P2_ calculated is equal to 3.7 × 10^13^ probe/cm^2^, taking into account experimental frequency change measured Δf (−180 Hz), QCM sensitivity s (350 pg/Hz), Avogadro constant N_A_ (6.023 × 10^23^ mol^−1^), QCM adsorption surface S (0.2 cm^2^) and probe molecular weight M_P2_ (6,370 g/mol). Probe adsorption surface 1/Γ_P2_ is equal to 2.7 nm^2^ per probe. This value, higher than 2.2 nm^2^ per probe reported for a similar sensor design [6], depends on adsorption surface roughness, as it was shown elsewhere [24]. The second step allows the selectivity to be checked: no frequency changes are measured during flowing of a 20 µg/mL random strand (R) HEPES solution, meaning that the there is no hybridization or non-specific adsorption of a non complementary strand. The last step, hybridization, was performed by flowing of a 20 µg/mL complementary target (T2) HEPES solution. The frequency change measured was −86 Hz. The hybridization efficiency η_T2_ (%) of hybridized targets T2 *versus* grafted probes P2 is:

η_T2_ = 100∙Δf_T2_∙M_P2_/Δf_P2_∙M_T2_       (2)

η_T2_ is equal to 40%, taking into account the molecular weights M of probe (P2) (6,370 g/mol) and target (T2) (6,055 g/mol), and frequency changes Δf during circulation of probe (P2) (−225 Hz) and target (T2) (−86 Hz) solutions. Four biosensors were designed on different quartz samples to check the reproducibility: probe surface density Γ_P1_ is (3.7 ± 0.5) × 10^13^ probe/cm^2^, hybridization ratio η_Τ2_ 42 ± 2%, frequency change during complementary target hybridization Δf_T2_ is 89 ± 13 Hz and half-time hybridization reaction 149 ± 6 s. The 42% hybridization ratio efficiency measured is close to 44% reported for a QCM DNA-biosensor designed by grafting a disulfide-labeled probe on a gold surface [25].

### 3.3. Biosensor Designed with Biotin-Labeled Probe

**Figure 4:**
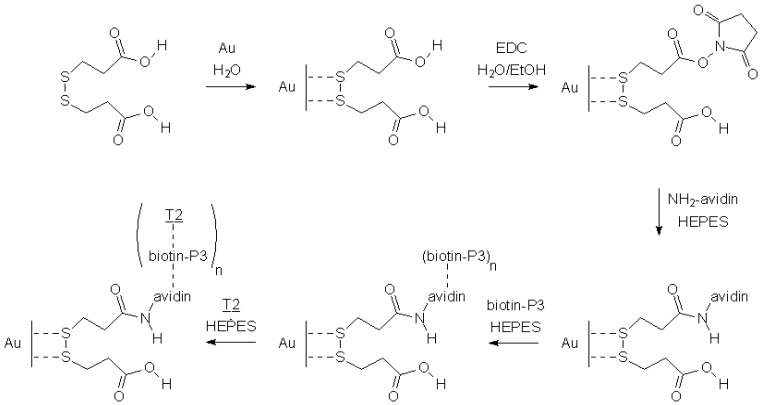
Avidin grafting, biotin-labeled probe (P3) grafting and target (T2) hybridization. n is the mean number of biotin per avidin (0 < n < 4).

Grafting of biotin-labeled probe (P3) on the gold quartz surface and complementary target (T2) recognition is achieved in five steps (Figure 4).

**Figure 5:**
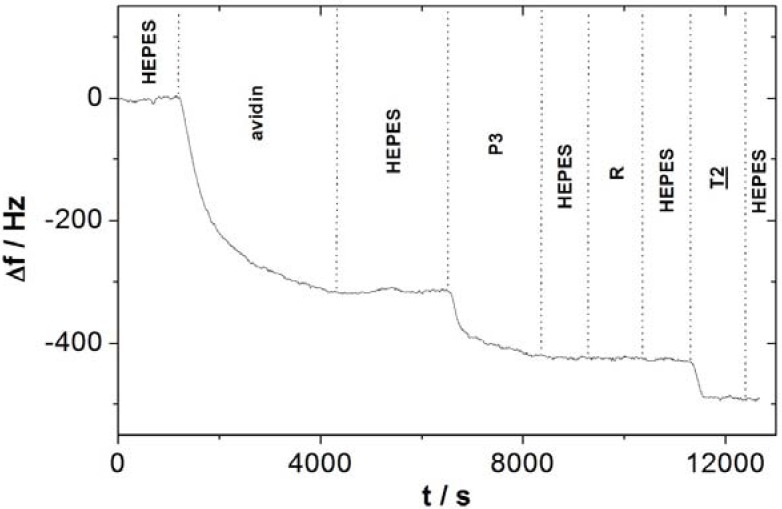
Microbalance frequency changes during avidin grafting, biotin-labeled probe (P3) grafting and selective complementary target (T2) recognition.

The frequency was not recorded during formation of the self-assembled dithiopropionic acid and EDC ester activation, due to the low molecular weight of these compounds, being too feeble for detection with a 27 MHz quartz crystal microbalance sensitivity. Frequency changes during avidin grafting, biotin-labeled probe (P3) grafting and complementary target (T2) recognition, are presented in Figure 5.

The first step, avidin grafting, was performed by circulating a 10 mg/mL avidin solution. The frequency change measured Δf_avidin_ is −316 Hz. The avidin surface density Γ_avidin_ (avidin/cm^2^) is equal to:

Γ_avidin_ = |Δf_avidin_|∙s∙N_A_/S∙M_avidin_       (3)

Γ_avidin_ calculated is equal to 4.9 × 10^12^ avidin/cm^2^, taking into account frequency change Δf_avidin_ measured (−316 Hz), QCM sensitivity s (350 pg/Hz), Avogadro constant N_A_ (6.023 × 10^23^ mol^−1^), QCM active surface S (0.2 cm^2^) and avidin molecular weight M_avidin_ (68,000 g/mol). The grafting of the probe was performed by circulating a 20 µg/mL biotine-labeled probe (P1') NaCl solution. The frequency change measured during this step was −105 Hz. The mean value n of biotin-labeled probe (P3) complexes per grafted avidin on the surface is:

n = Δf_P3_∙M_avidin_/Δf_avidin_∙M_P3_       (4)

n is equal to 3.5 taking into account the molecular weight M of probe (P3) (6,472 g/mol), of avidin (68,000 g/mol), frequency changes Δf during circulation of probe (P3) (−105 Hz) and avidin (−316 Hz) solutions. This 3.5 mean value is close to the maximum number of biotin per avidin molecule in the bulk solution complex, which is equal to four. The difference could be explained by steric hindrance in the biolayer. The probe P3 surface density Γ_P3_ (probe/cm^2^) is equal to:

Γ_P3_ = n∙Δ_avidin_       (5)

Γ_P3_ calculated is equal to 1.7 × 10^13^ probe/cm^2^. The third step allows the selectivity to be checked. No frequency change was measured during flowing of a 20 µg/mL random strand (R) HEPES solution, meaning that the there is no hybridization or non-specific adsorption of a non complementary strand. The last step, hybridization, was performed by flowing of a 20 µg/mL complementary target (T2) HEPES solution. The frequency change measured was −62 Hz. The hybridization ratio efficiency η _T2_ of hybridized targets *versus* grafted probes is:

η_T2_ = 100∙Δf_T2_∙M_P3_/Δf_P3_∙M_T2_       (6)

η_T2_ measured is equal to 63%, taking into account the molecular weight M of probe (P3) (6,472 g/mol) and target (T2) (6,055 g/mol), and frequency changes Δf during circulation of probe (P3) (−105 Hz) and target (T2) (−62 Hz) solutions. Three biosensors were designed on different quartz to check the reproducibility: number of biotin per avidin is 3.0 ± 0.6, hybridization ratio efficiency is 79 ± 15%, frequency change during hybridization step is −61 ± 8 Hz and half-time of biosensor hybridization reaction is 101 ± 16 s.

### 3.4. Comparison of Biosensor Characteristics and Behaviors

Characteristics and behaviors of biosensors designed with thiol and biotin labeled probes are presented in Table 2. Half-time t_1/2_ hybridization was calculated by subtracting 25% hybridization time from 75% hybridization time:

t_1/2_ = t_3/4_ − t_1/4_       (7)

**Table 2:** Biosensor grafting and hybridization characteristics and behaviors: oligonucleotide label (5'); number n of biosensors designed; probe density Γ(probe∙cm^−2^) on the gold covered quartz surface; hybridization efficiency η(%); quartz frequency change Δf (Hz) during DNA target hybridization and half-time t_1/2_(s) of biosensor hybridization reaction.

biosensor		grafting	hybridization		
DNA label	n	Г/probe.cm^−2^	η/%	|∆f|/Hz	t_1/2_/s
thiol	4	(3.7 ± 0.5) × 10^13^	42 ± 2	89 ± 13	149 ± 26
biotin	3	(1.4 ± 0.3) × 10^13^	79 ± 15	61 ± 8	101 ± 16

Biotin-labeled probes more diluted on the surface are better accessible, in comparison with thiol-labeled ones, resulting in a faster and more efficient hybridization. DNA target concentrations of this study are equal to 20 µg/mL. It was recently shown that hybridization efficiency at room temperature is poorly dependent on DNA target concentration in the range from 100 pg/mL to 20 µg/mL [26]. The frequency change during hybridization, related to mass sensitivity, is higher in the case of the biosensor designed with a thiol probe, due to the higher probe surface density. These results are consistent with a recent study, on the design of QCM DNA biosensor with thiol- and biotin-labeled probes, for the diagnosis of a fish pathogenic virus: RNA recognition is more efficient with the biosensor designed with a biotin probe [18]. Regeneration of biosensors with an alkaline saline solution (NaOH 0.5 M, NaCl 3 M) was achieved *in fine*. There is a loss of 16% of hybridization efficiency for a second hybridization run in the case of the biosensors designed with the thiol-labeled probe and of 26% with the biotin-labeled ones. A biosensor designed with biotin-labeled probes is less resilient due do avidin denaturation in alkaline saline medium.

## 4. Conclusions

Both thiol- and biotin-labeled probes can be used to design reproducible biosensors of the oligonucleotide target encoding for the large ribosomal RNA sub-unit of the microalga *Alexandrium minutum*, which is responsible for paralytic shellfish poisoning (PSP) on the European and Asian coasts. Kinetic quartz crystal microbalance frequency curves indicate that the thiol-based biosensor is more sensitive and resilient to denaturation and that the biotin-based one has a shorter time response and a higher hybridization efficiency.
